# Biovalorisation of crude glycerol and xylose into xylitol by oleaginous yeast *Yarrowia lipolytica*

**DOI:** 10.1186/s12934-020-01378-1

**Published:** 2020-06-03

**Authors:** Ashish A. Prabhu, Dominic J. Thomas, Rodrigo Ledesma-Amaro, Gary A. Leeke, Angel Medina, Carol Verheecke-Vaessen, Frederic Coulon, Deepti Agrawal, Vinod Kumar

**Affiliations:** 1 School of Water, Energy and Environment, Cranfield University, Cranfield, MK43 0AL UK; 2 Department of Bioengineering and Imperial College Centre for Synthetic Biology, Imperial College London, London, SW7 2AZ UK; 3 School of Chemical Engineering, University of Birmingham, Birmingham, B15 2TT UK; 4 Biochemistry and Biotechnology Area, Material Resource Efficiency Division, CSIR-Indian Institute of Petroleum, Mohkampur, Dehradun, 248005 India

**Keywords:** Glycerol, Xylose, *Yarrowia lipolytica*, Biotransformation, Xylitol

## Abstract

**Background:**

Xylitol is a commercially important chemical with multiple applications in the food and pharmaceutical industries. According to the US Department of Energy, xylitol is one of the top twelve platform chemicals that can be produced from biomass. The chemical method for xylitol synthesis is however, expensive and energy intensive. In contrast, the biological route using microbial cell factories offers a potential cost-effective alternative process. The bioprocess occurs under ambient conditions and makes use of biocatalysts and biomass which can be sourced from renewable carbon originating from a variety of cheap waste feedstocks.

**Result:**

In this study, biotransformation of xylose to xylitol was investigated using *Yarrowia lipolytica,* an oleaginous yeast which was firstly grown on a glycerol/glucose for screening of co-substrate, followed by media optimisation in shake flask, scale up in bioreactor and downstream processing of xylitol. A two-step medium optimization was employed using central composite design and artificial neural network coupled with genetic algorithm. The yeast amassed a concentration of 53.2 g/L xylitol using pure glycerol (PG) and xylose with a bioconversion yield of 0.97 g/g. Similar results were obtained when PG was substituted with crude glycerol (CG) from the biodiesel industry (titer: 50.5 g/L; yield: 0.92 g/g). Even when xylose from sugarcane bagasse hydrolysate was used as opposed to pure xylose, a xylitol yield of 0.54 g/g was achieved. Xylitol was successfully crystallized from PG/xylose and CG/xylose fermentation broths with a recovery of 39.5 and 35.3%, respectively.

**Conclusion:**

To the best of the author’s knowledge, this study demonstrates for the first time the potential of using *Y. lipolytica* as a microbial cell factory for xylitol synthesis from inexpensive feedstocks. The results obtained are competitive with other xylitol producing organisms.
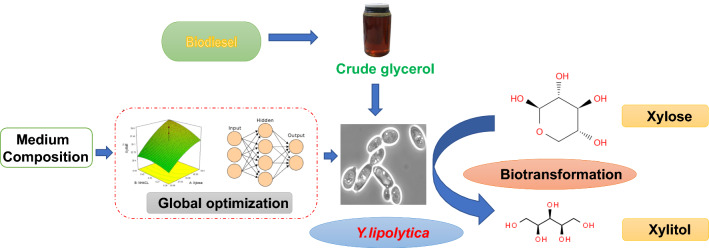

## Background

The development of green technologies is highly desired to stabilise the progressive depletion of non-renewable fossil-derived chemicals [1]. The bio-refinery concept is the promising green and sustainable approach to supplement the use of fossil-derived feedstocks, where a carbon reservoir present in the biomass is exploited for the integrated production of chemicals, fuels and energy. First generation biorefineries that use edible biomass feedstocks are well established. The food versus fuel debate gave rise to second generation biorefineries based on non-edible feedstocks and biomass wastes [2, 3]. These feedstocks have drawn significant attention as they can generate high value products from a renewable carbon source. Over the last two decades, there has been a large amount of research dedicated to the development of bioprocesses based on agro-industrial wastes [4].

Lignocellulosic biomass (LCB) is the most abundant biomass on earth and has three major components namely cellulose (34-50%), hemicellulose (19–34%) and lignin (11–30%). Hemicellulose, a hetero-polysaccharide consists largely of xylose (~ 90%), which contributes to 15–35% of the total dry cell weight of LCB [5, 6]. Despite being the second most abundant sugar after glucose, xylose valorisation through biotechnological routes is often overlooked. Most of the microbeslack xylose assimilation pathways as xylose is rarely present in the environment and in free form in natural products. If the pathways exist, it is not a preferred carbon source and its uptake is suppressed in the presence of glucose due to carbon catabolite repression [7]. These challenges make pentose sugar a lesser lucrative option as an exploitable carbon source. However, efficient utilisation of xylose is essential for the commercial viability of lignocellulosic biorefineries [8, 9]. Like xylose/LCB, crude glycerol (CG) is another waste product of interest as a carbon source for microbial conversion. CG is major by-product of many industrial processes, such as bioethanol, oleochemical, chemical and biodiesel. The rapid growth in the biodiesel industry has led to an increase in CG production [10] with ~ 10 kg of CG production for every 100 kg of biodiesel. Biodiesel production increased exponentially from 2005 to 2015 and is forecasted to grow by another 35% by 2025 [11]. The surplus of biomass derived by-product streams like xylose and CG has emphasised the need to utilise them as carbon sources and avoid their disposal. It is therefore necessary to develop sustainable processes to transform these streams into promising value-added products.

Xylitol is a commercially important chemical. According to the US Department of Energy, xylitol is one of the top twelve platform chemicals, which can be produced from biomass [12]. Chemically, it is a polyol (sugar alcohol) containing five carbon atoms where a hydroxyl group is attached to each carbon atom. Xylitol is produced chemically/biochemically by the reduction of xylose. The molecule is equivalent to sucrose in sweetness but has less calories and lower glycaemic index [5, 13]. Due to its high endothermicity, low glycaemic rates, cariostatic properties, lack of carcinogenicity, non-involvement in the insulin metabolic pathway and non-interference with food nutritional value, xylitol has many applications in the food and pharmaceutical industries [14]. Xylitol has a large market and a 12% share of the total polyols market, which is expanding rapidly. The global market for xylitol in 2016 was 190,900 metric tons and is anticipated to reach 266,500 metric tons in 2022 with a value greater than US$ 1 billion [6].

*Yarrowia lipolytica* is a non-conventional, oleaginous, safe and robust yeast with multiple biotechnological applications. It has versatile characteristics such as high cell density cultivation, metabolic flexibility and tends to accumulate a wide array of industrially important metabolites. In addition, the yeast is non-pathogenic and has a GRAS (generally regarded as safe) status [15]. The unique features of *Y. lipolytica* make it a promising cell factory for the production of value-added chemicals. Glycerol is the most preferred carbon source for *Y. lipolytica* and can metabolize it with great efficiency [16–18]. According to the literature, the majority of the *Yarrowia* strain cannot grow on xylose as they possess strong xylose reductase activity but have low xylitol dehydrogenase activity, however, some can biotransform xylose into xylitol [19, 20].

The current study was undertaken to investigate the xylitol accumulating ability of *Y. lipolytica* Po1t (Ura^+^, Leu^+^) [19]. The biotransformation of xylose into xylitol was carried out by growing *Y. lipolytica* on pure glycerol (PG), CG as well as glucose The work studies the screening of co-substrates for cell growth, media optimization in a shake flask, scale up in bioreactor and downstream processing of xylitol. This study is the first to demonstrate the potential of using *Y. lipolytica* as a microbial cell factory for xylitol synthesis from inexpensive feedstocks.

## Results

### Shake flask screening of co-substrates for growth of *Yarrowia lipolytica* and subsequent biotransformation of xylose to xylitol

**Fig. 1:**
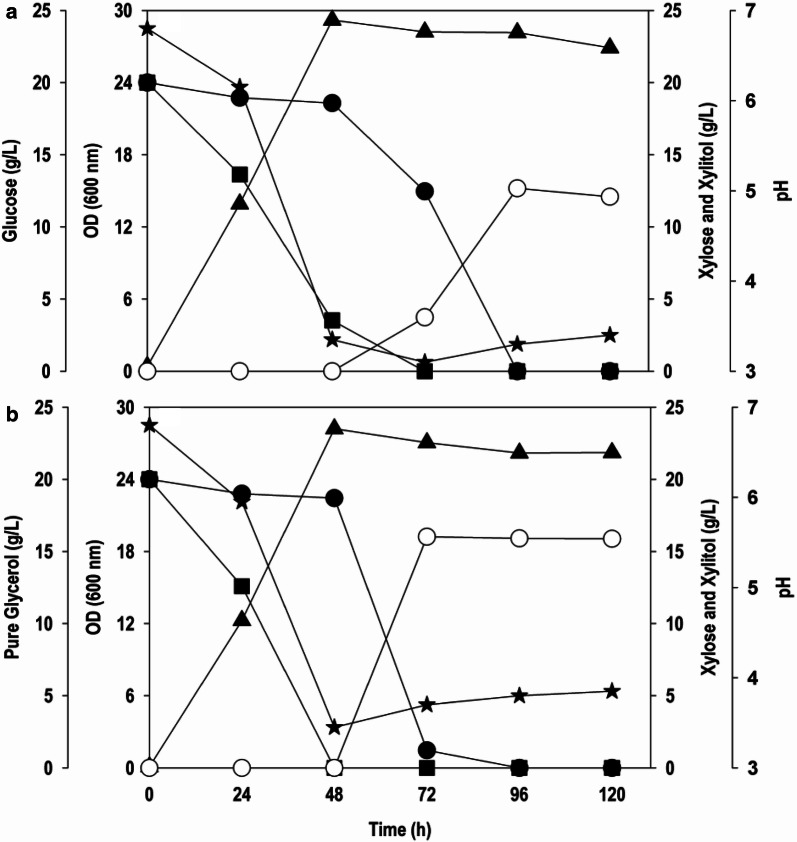
Time course profiles of *Y. lipolytica* on; **a** glucose and xylose, **b** PG and xylose. Symbols: filled square (glucose or PG), filled circle (xylose), filled triangle (OD_600_), empty circle (xylitol) and filled star (pH)

The biotransformation of xylose to xylitol using *Y. lipolytica* cell factory was evaluated under shake flask conditions. The *Y. lipolytica* Po1t (Ura^+^ Leu^+^) strain used in this study can transform xylose into xylitol but it cannot grow on xylose as a sole carbon source (data not shown). Two carbon sources namely glucose and glycerol were evaluated for biomass accumulation of *Y. lipolytica* cells and subsequent biotransformation of xylose into xylitol. The growth profile of *Y. lipolytica* clearly indicated that the assimilation of glycerol was faster than that of glucose as shown by Fig. 1. The yeast was able to consume 20.0 g/L of glycerol within 48 h whereas more than 15% of glucose was left unconsumed in the same time and complete glucose consumption was evident by 72 h. The highest OD_600_ values obtained for glycerol and glucose were quite similar, 29.2 and 28.0, respectively. After the majority of glucose or glycerol was exhausted (~ 48 h), biotransformation began and in the next 24–48 h, the maximum production of xylitol was recorded. However, owing to earlier consumption of glycerol than glucose, the biotransformation rate was significantly faster in the former case than the later and the maximum xylitol production was achieved in 72 h for glycerol compared to 96 h for glucose. Using glycerol as a co-substrate, the xylitol titre was 16.0 g/L with a yield of 0.80 g/g, however, when glucose was used as a co-substrate the highest xylitol titer and conversion yield were 12.7 g/L and 0.64 g/g, respectively. The pH decreased during the growth phase, reducing below 4.0 after 48 h and then remained almost constant during the xylitol production phase. The biotransformation yield obtained was higher with glycerol and was therefore selected as the co-substrate for further experiments.

### Media optimization in shake flask to maximize biotransformation of xylose to xylitol

#### Central composite design (CCD)

Statistical methods measure the effects of changing operating variables and their mutual interactions on the process through experimental design techniques. In the present study, the central composite design (CCD) was employed to determine the optimum value of media components (xylose, YNB, NH_4_Cl and phosphate buffer) to maximize xylitol production in shake flask while keeping glycerol concentration constant. The design matrix and the corresponding results of observed and predicted responses (xylitol titre) are shown in Table 1. The experimental run 8 resulted in the production of 37.2 g/L xylitol for a 50.0 g/L initial concentration of xylose (Table 1). However, the best run in terms of xylitol yield was found for run 9 where 0.95 g_xylitol_/g_xylose_ was produced for an initial xylose concentration of 20.0 g/L.

**Table 1:** CCD and ANN design matrix of variables with experimental and predicted response for xylitol production

Experiment no.	Xylose (g/L)	NH_4_Cl (w/v %)	YNB (w/v %)	Phosphate buffer (mM)	Xylitol (g/L)	CCD predicted	ANN predicted
1	20	0.2	0.2	35	17.47	18.04	18.79
2	50	0.2	0.2	35	22.20	22.80	22.48
3	20	0.5	0.2	35	13.11	11.81	14.99
4	50	0.5	0.2	35	20.05	19.51	20.45
5	20	0.2	0.5	35	10.20	10.52	4.83
6	50	0.2	0.5	35	21.77	22.48	19.70
7	20	0.5	0.5	35	18.70	20.81	18.92
8	50	0.5	0.5	35	37.22	35.73	33.50
9	20	0.2	0.2	100	18.99	26.02	23.06
10	50	0.2	0.2	100	29.53	27.94	30.42
11	20	0.5	0.2	100	17.85	17.65	19.66
12	50	0.5	0.2	100	23.30	22.52	23.14
13	20	0.2	0.5	100	4.74	5.79	5.90
14	50	0.2	0.5	100	14.07	14.91	13.92
15	20	0.5	0.5	100	15.00	13.95	14.27
16	50	0.5	0.5	100	26.08	26.03	31.47
17	35	0.35	0.35	67.5	20.71	24.25	24.96
18	35	0.35	0.35	67.5	27.18	24.25	24.96
19	35	0.35	0.35	67.5	24.53	24.25	24.96
20	35	0.35	0.35	67.5	24.84	24.25	24.96
21	5	0.35	0.35	67.5	20.57	19.33	21.37
22	65	0.35	0.35	67.5	34.98	36.16	37.22
23	35	0.05	0.35	67.5	12.41	10.67	11.56
24	35	0.65	0.35	67.5	13.88	15.55	15.66
25	35	0.35	0.05	67.5	29.69	30.82	29.96
26	35	0.35	0.65	67.5	28.00	26.80	23.25
27	35	0.35	0.35	2.5	23.62	23.15	23.75
28	35	0.35	0.35	132.5	21.03	21.43	21.08
29	35	0.35	0.35	67.5	24.87	27.46	24.96
30	35	0.35	0.35	67.5	29.78	27.46	24.96

**Table 2:** Analysis of variance for xylitol production

Source	DF	Seq SS	Adj SS	Adj MS	F	p
Blocks	1	68.61	68.61	68.61	15.35	0.002
Regression	14	1402.25	1402.25	100.16	22.41	< 0.001
Linear	4	489.28	489.28	122.32	27.37	< 0.001
Xylose	1	424.91	424.91	424.91	95.08	< 0.001
NH_4_Cl	1	35.71	35.71	35.71	7.99	0.01
YNB	1	24.22	24.22	24.22	5.42	0.04
Phosphate	1	4.44	4.44	4.44	0.99	0.37
Square	4	404.82	404.82	101.21	22.65	< 0.001
Xylose*Xylose	1	9.54	0.14	0.14	0.03	0.86
NH_4_Cl*NH_4_Cl	1	341.93	352.75	352.75	78.93	< 0.001
YNB*YNB	1	7.65	3.14	3.14	0.7	0.42
Phosphate*Phosphate	1	45.7	45.7	45.70	10.23	0.01
Interaction	6	508.15	508.15	84.69	18.95	< 0.001
Xylose*NH_4_Cl	1	8.73	8.73	8.73	1.95	0.18
Xylose*YNB	1	51.99	51.99	51.99	11.63	0.004
Xylose*Phosphate	1	8.09	8.09	8.09	1.81	0.2
NH_4_Cl*YNB	1	273.24	273.24	273.24	61.14	<0.001
NH_4_Cl*Phosphate	1	4.56	4.56	4.56	1.02	0.33
YNB*Phosphate	1	161.56	161.56	161.56	36.15	< 0.001
Residual Error	14	62.57	62.57	4.47		
Lack-of-Fit	10	29.03	29.03	2.90	0.35	0.92
Pure Error	4	33.54	33.54	8.38		
Total	29	1533.43				

DF: degree of freedom; Seq SS: sequential sum of square; Adj SS: adjusted sum of square; Adj MS: adjusted mean square; F: variance ratio (Fisher F-value); p: probability value

The results were analysed using the Analysis of Variance (ANOVA) shown in Table 2. The error term, which indicates that the amount of variation in the response data, is very low. According to the ANOVA, the regression model for the xylitol production showed high significance with a Fisher’s F value of 22.41 and explains most of the variations present in the experimental design [21]. The “p” value of 0.92 for lack of fit indicated that the response for xylitol concentration was not significant relative to the pure error. The correlation coefficient (R^2^) between the experimental and model-predicted values of response variables showed high statistical significance of 94.16%, which implies that only 5.84% of the total variation was not explained by the model. The Student’s t distribution and the corresponding p values shows that most of the interaction terms are statistically significant (P < 0.05), except for the interaction terms involving xylose with NH_4_Cl and phosphate buffer which showed insignificance. The second-order polynomial equation for xylitol production by CCD is given in Eq. (1).

1 $$\begin{aligned} {\text{Y}}_{\text{xylitol}} &= 25.90 + 4.45{\text{X}}_{1} + 1.46{\text{X}}_{2} - 0.75{\text{X}}_{3}\\&\quad  - 0.68{\text{X}}_{4} + 0.008{\text{X}}_{1}^{2} - 3.64{\text{X}}_{2}^{2} + 0.27{\text{X}}_{3}^{2} \\&\quad - 1.35{\text{X}}_{4}^{2} + 0.36{\text{X}}_{1} {\text{X}}_{2} + 1.42{\text{X}}_{1} {\text{X}}_{3} \\&\quad - 0.33{\text{X}}_{1} {\text{X}}_{4} + 3.7{\text{X}}_{2} {\text{X}}_{3} - 0.15{\text{X}}_{2} {\text{X}}_{4} \\&\quad - 2.8{\text{X}}_{3} {\text{X}}_{4} \\ \end{aligned}$$

where Y is the xylitol concentration (g/L) and X_1_, X_2_, X_3_ and X_4_ are xylose (g/L), YNB (w/v  %), ammonium chloride (w/v  %) and phosphate buffer (mM), respectively.

**Fig. 2:**
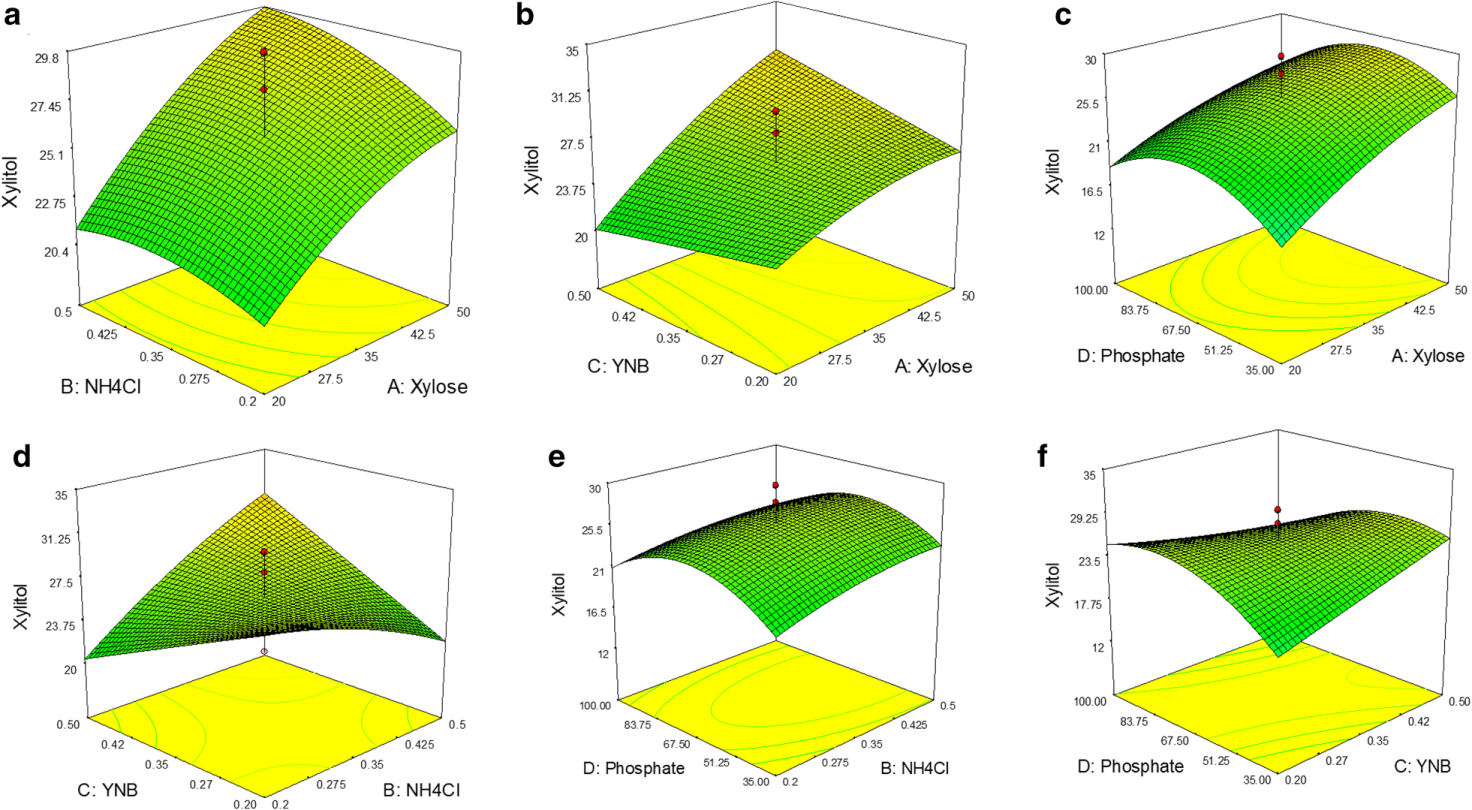
Three-dimensional response surface plot for xylitol production showing the interactive effects of **a** xylose & NH_4_Cl, **b** xylose & YNB, **c** xylose & phosphate buffer, **d** YNB & NH_4_Cl, **e** phosphate buffer & NH_4_Cl and **f** YNB & phosphate buffer

The response surface plot for the interaction between the media components is shown in Fig. 2. The 3D surface plot gives an overview of the interaction between the two components on xylitol production by keeping the other parameters at central values. The interaction between xylose and NH_4_Cl showed a positive effect on xylitol production and a progressive increment in the xylitol titer was observed with the increasing concentration of both components. Further, higher xylose concentrations caused a steep reduction in xylitol titer. Similarly, the interactions between xylose & YNB (Fig. 2b) and NH_4_Cl & YNB (Fig. 2d) showed positive effects (P < 0.05) on xylitol production indicating that higher concentrations of YNB and NH_4_Cl will lead to the enhanced production of xylitol. On the other hand, the interaction between xylose & phosphate buffer (Fig. 2c), phosphate buffer & NH_4_Cl (Fig. 2e) and YNB & phosphate buffer (Fig. 2f) showed statistical insignificant values, which indicate one of the components has to be kept at a minimum to enhance the xylitol production.

### Optimization of process parameters using artificial neural network linked genetic algorithm (ANN-GA)

**Fig. 3:**
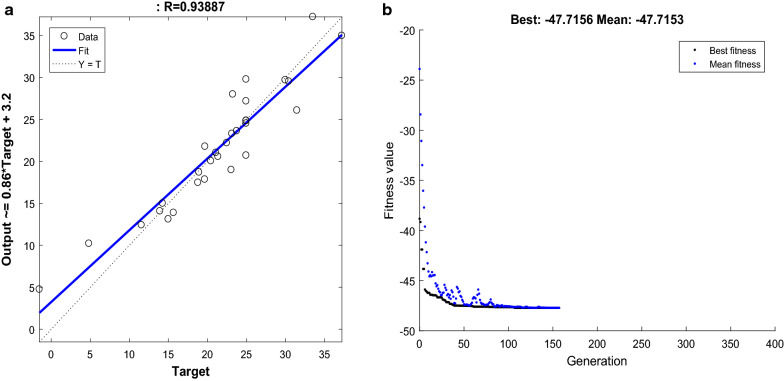
The prediction performance of ANN models for xylitol production: **a** overall regression value of simulated ANN; **b** best and average fitness values with successive generations showed gradual convergence to the optimum value

The experimental design generated by the CCD was used as input feed for ANN algorithm. The overall data set were divided into three subsets: training (20 data points), validation (5 data points) and test sets (5 data points). The training was carried for 1000 epochs, the mean square error (MSE) and R^2^ value for data set involved in xylitol production are shown in Additional file 1: Table S1. The data points apart from the training are used to examine the validation. During training the data over fits and substantial error will be accumulated on the validation. When the error on the validation reaches the threshold point the weights and biases are adjusted to minimize the error [22, 23]. Network topology have a crucial role in predicting results, the input–output neuron of ANN is the resemblance of input and output data used in this study. The number of neurons in the hidden layer was determined by trial and error method to minimize MSE. The MSE of xylitol production was found to be 7.425. The predicted value of ANN for xylitol is shown in Table 1. The optimum value was achieved with 4 inputs, 8 hidden layers and 1 output layer. The simulation of ANN resulted in a R^2^ of 0.938 between the actual experimental production values (Fig. 3a). In order to further optimize the solution space for global optimum, the genetic algorithm (GA) was adapted to train ANN values. The values of GA specific parameters used in the optimization technique were as follows: population size = 20, cross over probability = 0.8, mutation probability = 0.01, No. of generation = 100. The maximum xylitol production of 47.7 g/L was observed with 160 iterations. The best fitness plot of the GA for xylitol production (Fig. 3b) maps the gradual convergence of the best fitness values of successive generations towards the final optimum value. The optimum values were found to be as following: xylose—55.0 g/L, NH_4_Cl—3.94 g/L, YNB—5.0 g/L and phosphate buffer—132.5 mM.

### Model validation under shake flask conditions

**Fig. 4:**
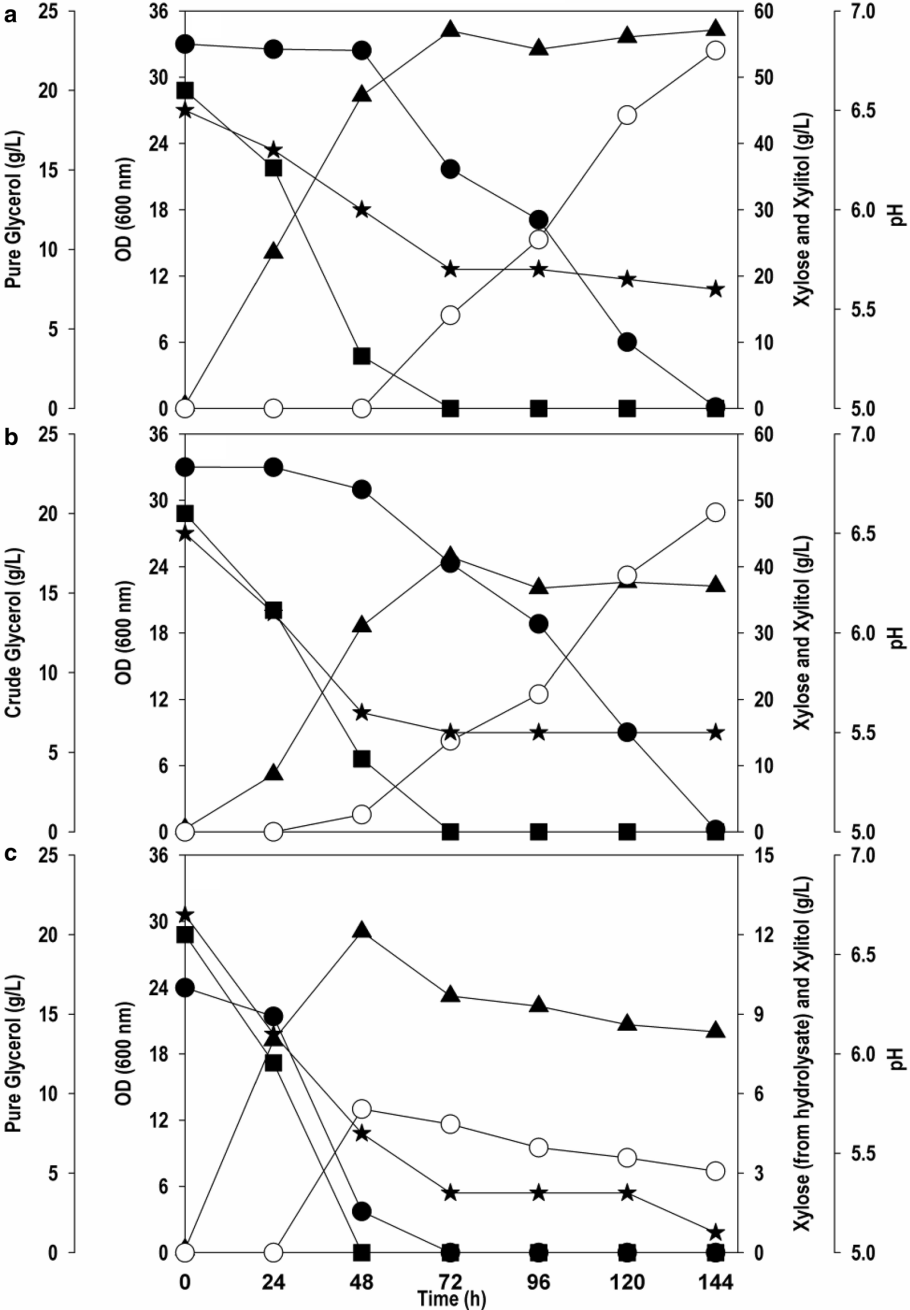
Shake flask cultivation of *Y. lipolytica* using optimized media on; **a** PG and xylose, **b** CG and xylose, **c** PG and xylose from SCB hydrolysate. Symbols: filled square (PG or CG), filled circle (xylose), filled triangle (OD_600_), empty circle (xylitol) and filled star (pH)

The validation experiments were performed in shake flasks based on the global optimum values obtained by ANN-GA training. Three different sets of experiments were conducted: PG + xylose (Fig. 4a); CG + xylose (Fig. 4b); PG + crude xylose (Fig. 4c). The crude carbon sources were included to test the ability of the *Y. lipolytica* strain to tolerate, utilize and valorise crude renewable sources. The glycerol uptake rate was similar for the case of PG and CG and the major fraction of glycerol carbon was depleted in first 48 h. The cell concentration obtained with PG (OD_600_: 34.2) was higher than CG (OD_600_: 24.9). The low cell OD_600_ in comparison to PG might be attributed due to the presence of some inhibitory components such as methanol present in the CG [24]. The results showed significant improvements in xylitol titer and yield in comparison to unoptimized composition. For the case of PG and xylose, 98% of xylose was transformed into xylitol, and a xylitol concentration of 54.0 g/L was achieved. On the other hand, for the co-fermentation of CG and xylose, a xylitol titre of 48.2 g/L was obtained with a conversion yield of 0.88 g/g. This difference could be attributed to the composition of CG. The higher buffer concentration of optimized medium suppressed the reduction in pH, and therefore, after an initial drop, the pH was stable around 5.5. The pH plays a crucial role in the transportation of xylose across the membrane [25].

The ability of *Y. lipolytica* to produce xylitol from crude xylose was also tested. For this, crude xylose obtained after hydrothermal pretreatment of sugarcane bagasse was used. Lignocellulosic material often contains compounds such as phenols, furan derivatives and aliphatic acids in large amounts which tend to inhibit microbial growth [26]. However, the cell growth (OD_600_: 29.1) was faster and almost unaffected by the presence of impurities/inhibitory molecules in crude xylose. The higher cell growth could also be contributed by other sugars such as glucose in the sugarcane bagasse (SCB) hydrolysate [27]. The conversion of crude xylose was 54% with the xylitol titre of 5.4 g/L. The low conversion yields obtained indicating the effect of impurities on the biotransformation ability of *Y. lipolytica.* A plausible reason for the low biotransformation yield from the xylose-rich SCB hydrolysate when compared to pure xylose could be due to its direct use without detoxification. Generally hydrothermal pretreatment leads to hydrolysis of acetyl groups attached to the hemicellulosic backbone and as a result acetic acid is formed [28]. It is likely that acetic acid and other furan aldehydes were inhibitory and their presence negatively affected the performance of *Y. lipolytica* during biotransformation. This experiment also gave an insight into the importance of undertaking a detoxification step to remove or minimize the inhibitory compounds.

### Submerged batch cultivations in bioreactor

**Fig. 5:**
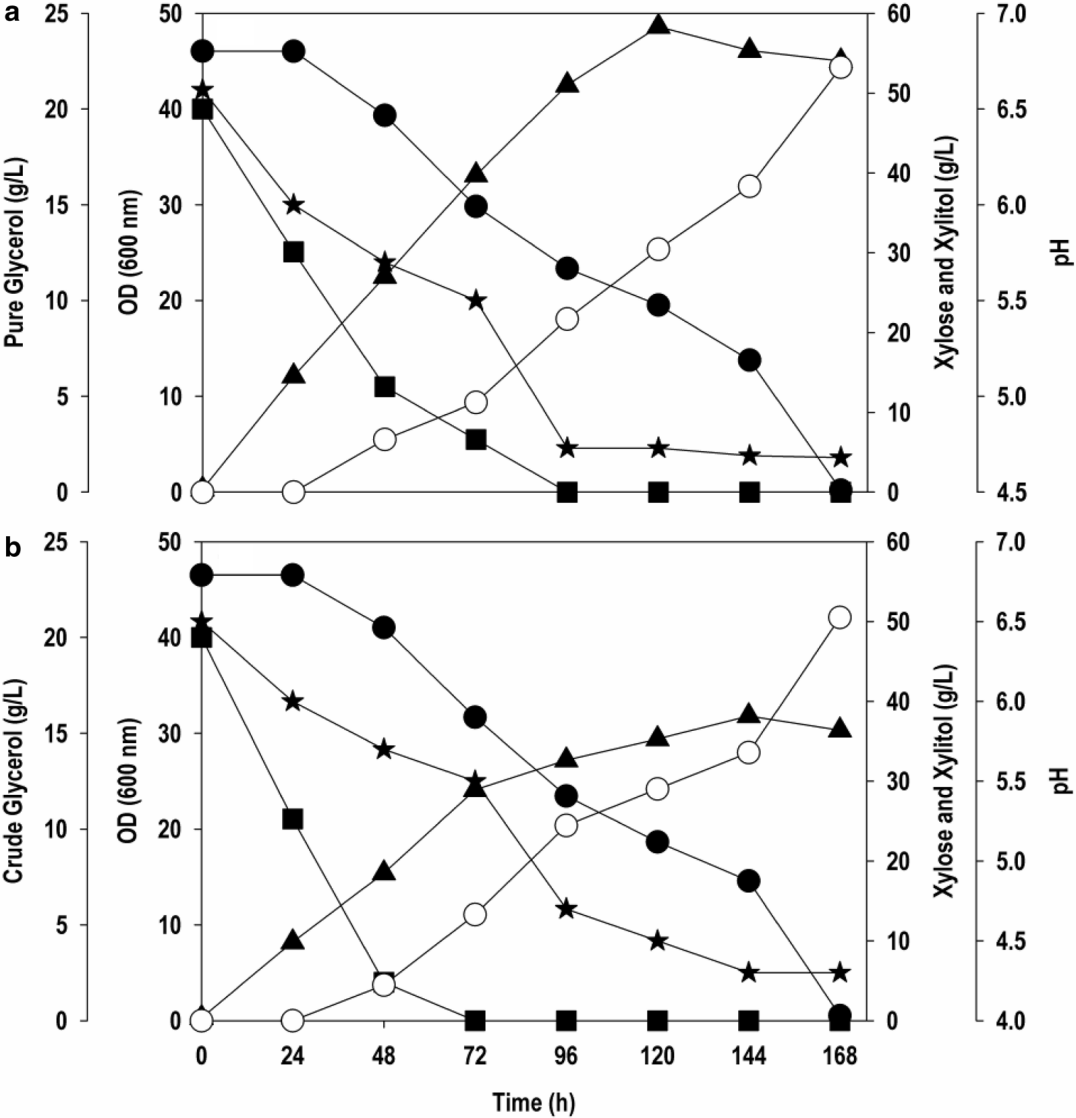
Batch kinetics of substrate assimilation, cell growth, pH and xylitol formation by *Y. lipolytica* in bioreactor during co-fermentation on; **a** PG and xylose, **b** CG and xylose. Symbols: filled square (PG or CG), filled circle (xylose), filled triangle (OD_600_), empty circle (xylitol) and filled star (pH)

In order to scale up the fermentation and validate the optimized medium composition, batch cultivations were carried out in a 2.5 L scale bench bioreactor with 1 L working volume. The process condition mimicked was exactly that of the shake flask studies except for the aeration. The aeration rate was maintained at 2.0 L/min for first 48 h and then reduced to 1.0 L/min for the rest of the test. Two separate batch fermentations were run with PG/xylose and CG/xylose. The time course profiles for both the fermentations were similar to that of the shake flask cultivations. Glycerol is the most preferred carbon source for *Y. lipolytica* and the presence of glycerol repressed the uptake of xylose as evident in Fig. 5. The gradual uptake of xylose concomitant with xylitol production was noticed when glycerol was almost completely exhausted. The maximum cell OD_600_ of 48.6 was observed at 120 h with co-fermentation of PG and xylose, which is higher than achieved during shake flask cultivation. By the end of 168 h there was complete consumption of xylose and a maximum 53.2 g/L xylitol (Fig. 5a) was produced with a yield of 0.97 g/g. The fermentation profile of *Y. lipolytica* with CG and pure xylose is shown in Fig. 5b. The maximum cell OD_600_ recorded was 31.8, not far from the value obtained in the shake flask studies. The yield of the xylitol was about 0.92 g/g with a titre of 50.5 g/L. Furthermore, pH for the fermentation using pure carbon source fluctuated between 6.5 and 4.7, whereas in the fermentation with CG the pH reduced to 4.3. It is plausible that CG had some impurities which interfered with the buffer capacity of the phosphate buffer, resulting in further drop in pH. In the growth phase (0–48 h) where biomass accumulation took place, aeration rate was high as glycerol assimilation is known to be dependent on the oxygen uptake rate [29]. Low aeration is maintained in the bioreactor during the xylitol production phase because excess aeration causes re-oxidation of NADH, a co-factor necessary for xylitol production from xylose and NAD^+^ produced can facilitate further metabolism of xylitol for cell growth [30].

### Xylitol production by resting cells

**Fig. 6:**
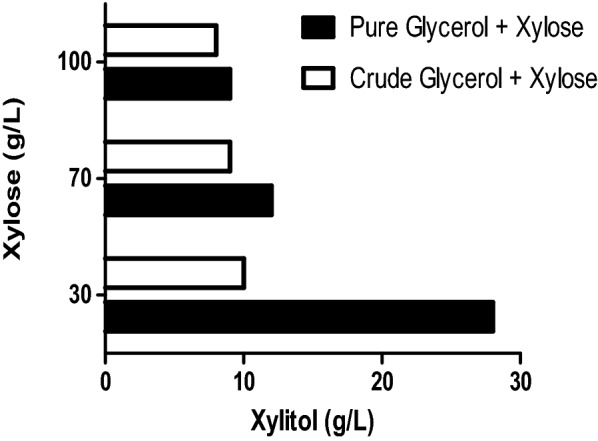
Biotransformation of xylose to xylitol by resting cells of *Y. lipolytica* grown using PG and CG

Resting cells are metabolically active non-growing cells [31]. Resting cells show an advantage over active cells such as simple operation, no requirement of nutrient medium and convenient downstream processing [32, 33]. In the current study *Y. lipolytica* is carrying out a single step biotransformation of xylose to xylitol. It is therefore worth exploring the potential of the yeast as a biocatalyst for the continuous production of xylitol through reusability of the cells; in other words, the growth phase was split from the xylitol production phase. The *Y. lipolytica* cells were grown using the PG and CG in shake flask using the culture medium as described in “Material and methods” section. The fermentation was terminated once the cell OD_600_ reached 20–25, nearly after 48 h and the cells were collected through centrifugation. The obtained cell pellet was suspended in buffer containing only xylose. The feasibility of using the resting cells of *Yarrowia lipolytica* for xylose biotransformation was checked by suspending the glycerol grown cell pellets in buffer containing three different concentrations of xylose (30, 70 and 100 g/L). It is evident from Fig. 6 that the conversion of xylose to xylitol was not satisfactory. The xylitol obtained for the case of cells accumulated on CG was ~ 10 g/L regardless of xylose concentration. The performance of the resting cells grown in PG were better and the highest amount of xylitol recorded with PG grown cell was ~ 28 g/L from 30 g/L xylose. The experimental results clearly indicated that the xylitol biotransformation yield significantly reduced with further increase in xylose levels. The probable cause for the cessation of the biocatalytic activity, may be due to the lack of ability of the already built biomass of *Y. lipolytica* cells to replenish the rate limiting cofactors for continuous and smooth biotransformation as there was no nutrient in the bioconversion stage to support cofactor supply. The presence of xylose alone in the media would not be sufficient to produce the cofactors required for the conversion.

### Purification of xylitol from the fermentation broth

Downstream processing costs are usually one of the major obstacles for the economical production of chemicals. Crystallization is the more commonly adopted method in the purification of polyols as it allows recovery of xylitol in a purified form in a single step. In terms of energy consumption, crystallization is less energy intensive when compared to a distillation process. Crystallization can be performed using various methods such as solvent evaporation, cooling and salting out [34]. Xylitol is a solid at room temperature with melting point > 90 °C. In the initial step, the coloured substance recovered was clarified using charcoal treatment, with 5% activated charcoal. The fermentation broth almost became translucent and the impurities such as residual xylose were removed. The recovery of xylitol after charcoal treatment step was 76.2 and 77.1% for CG/xylose and PG/xylose, respectively, as shown in Table 3. A subsequent alcohol precipitation step further reduced the recovery of xylitol. The final crystallization step carried out at − 20 °C for 72 h resulted in 35.3 and 39.5% xylitol recovery for CG/xylose and PG/xylose, respectively (Table 3). Despite the presence of impurities, similar recovery results were obtained with CG and PG. The results of xylitol recovery are encouraging and can be improved with further modifications.

**Table 3:** Xylitol titer and percent recovery obtained during different steps of downstream processing of fermentation broth from batch cultivation of *Y. lipolytica* in shake flask on CG/xylose and pure PG/xylose

Purification steps	CG + Xylose		PG + Xylose	
	Xylitol (g/L)	Recovery (%)	Xylitol (g/L)	Recovery (%)
Crude fermentation broth	48.2	100	54.0	100
Activated charcoal treatment	37.1	76.9	39.7	73.5
Alcohol precipitation	24.2	49.7	27.1	52.6
Crystallization	17.2	35.3	20.3	39.5

## Discussion

Xylitol is a polyol of commercial interest due to its high sweetening power and anticariogenic properties. Due to its multiple benefits, the market is growing with an increase of 6% per year. The industrial route requires pure xylose and the process is operated at high temperature and pressure. The chemical route is advantageous in terms of yield but has a number of hurdles to be overcome such as extensive purification, product recovery, catalyst deactivation, and energy intensity. All these factors make the process expensive and non-sustainable [14]. The biotechnological production of xylitol is environmentally safe and does not use toxic catalysts. The approach would be sustainable and create economic benefits if integrated with waste feedstocks rich in renewable carbon [13, 35]. The review of the literature shows that there has been two strategies for the bioproduction of xylitol; use of xylose as sole carbon source for cell growth as well as xylitol accumulation; and co-feeding another carbon source along with xylose for biomass formation. The co-fermentation of xylose and another substrate is preferred over the sole use of xylose for microbial xylitol production [36–38]. In the former approach, low yield and productivities are obtained due to a number of reasons; slow metabolism on xylose, partitioning of xylose flux between cell growth and xylitol formation, and re-consumption of produced xylitol. Therefore, it is more desirable to grow cell biocatalysts on a preferred carbon source so that a high cell density could be generated in a short time leading to higher productivities and yield.

**Fig. 7:**
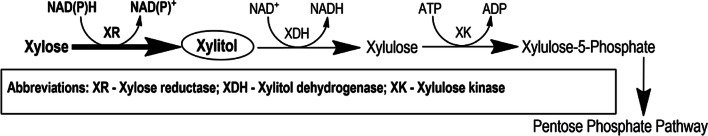
Xylose metabolism in yeast [5, 37]

The current study examined the potential of non-conventional yeast *Y. lipolytica* for xylitol production which is known to accumulate high levels of lipids, organic acids and polyols [15]. The biochemical production of xylitol takes place through reduction of xylose to xylitol mediated by xylose reductase (XR) and the electron transfer for this reaction is facilitated through participation of the redox cofactor NAD(P)H (Fig. 7). The produced xylitol is further oxidized to xylulose catalysed by xylitol dehydrogenase (XDH), which is then phosphorylated to xylulose-5-phosphate by enzyme xylulose kinase (XKS) and enters the central carbon metabolism for cell maintenance and growth. The absence of XDH enzyme, its weak activity and/or imbalance in activities of XR/XDH result in accumulation of xylitol [14, 37, 39]. The majority of *Y. lipolytica* strains cannot grow on xylose as documented by many literature reports [17, 19, 27]. According to Rodriguez et al. [20], the complete xylose pathway exists in *Y. lipolytica* but inability to grow robustly on xylose arises due to poor expression of key enzymes (XDH and XKS) controlling the pathway. The *Y. lipolytica* Po1t (Ura^+^ Leu^+^) strain used in the current study and in a previous work [19], has been shown to accumulate xylitol when cultured on xylose. The high yield obtained was a stimulus to carry out a detailed study to investigate the potential of *Y. lipolytica* for xylitol production.

Xylitol is an extracellular metabolite and its production is affected by many factors including medium composition, cell density and growth rate. Besides high xylose concentrations, an optimal balance of other nutrients is necessary to achieve industrial levels of xylitol [39, 40]. We started growing *Y. lipolytica* on a mixture of glucose or glycerol and xylose to choose better co-substrate. We obtained similar results for cell growth on both the carbon sources, however, glycerol assimilation was faster than that of glucose. In addition, the biotransformation rate and yield on glycerol were significantly higher than glucose (Fig. 1). Glycerol being a more reduced carbon source than traditional carbohydrates (glucose/sucrose/xylose) can provide better supply of reducing equivalents (NAD(P)H which is beneficial for xylitol formation [37, 41]. The preference of *Y. lipolytica* for glycerol is also well documented in literature. The yeast prefers glycerol over glucose and presence of glycerol but not that of glucose represses the uptake of other carbon sources including glucose in co-fermentations [16–18]. Workman et al. [16] found that glycerol assimilation was accompanied with higher oxygen uptake rates in comparison to glucose and maximum growth rate of *Y. lipolytica* on glycerol (0.30 h^−1^) was 25% higher than on glucose (0.24 h^−1^). The hypothesis for glycerol preference/repression like effects by *Y. lipolytica* is that its genome contain only one hexose transporter but three genes linked with glycerol transport. Lubuta et al. [18] performed RNA-Seq-based transcriptome analysis in *Y. lipolytica* and argued that the higher expression of several transporters could be potentially related to the phenotypic observation of glycerol preference. A similar kind of phenomenon can be envisaged in the present strain as well. In addition, being an oleaginous yeast, *Y. lipolytica*, can consume even the CG, a major industrial by-product with same the efficiency as PG [42].

The media optimization using CCD coupled with ANN-GA resulted in higher biotransformation efficiency, attaining a yield of more than 90% using PG and CG with xylose. Previously Pappu and Gummadi [43] adapted the ANN-GA model for optimizing process parameters such as pH, temperature and volumetric oxygen transfer co efficient *K*_*La*_ to enhance xylitol production in *Debaryomyces nepalensis*. With hybrid ANN-GA optimization, they reported an optimum predicted error of 3.5% and maximized xylitol yield of 0.53 g/g in batch bioreactor. The results highlight the importance of nonlinear modelling to optimize parameters in biochemical systems. In our study, nitrogen source (NH_4_Cl) and YNB showed a momentous effect on the xylitol bioconversion. NH_4_Cl plays a crucial role in enhancing the protein/enzyme expression level as the transcription of carbon metabolising gene is relied upon as the nitrogen source [44]. On the other hand, YNB comprises of essential components such as amino acids, vitamins, salts and trace elements required for yeast growth. Xia et al. [45], reported excellent fermentation capability of xylose by *C. shehatae* in medium supplemented with YNB, whereas impaired growth was witnessed when the medium was devoid of YNB supplementation. In a recent work, YNB was found to be a significant component for xylitol production by *Candida tropicalis* JA2, when Plackett–Burman design was done for nitrogen sources and various salts [46]. The results of the shake flask were replicated when the experiments were scaled up in bioreactor. The fermentation profiles can be divided into two phases; growth and biotransformation phase. The biotransformation of xylose started only after when a large fraction of co-substrate was consumed and may be due to carbon catabolite repression. The interesting observation was that cell growth was continuously increasing even after complete consumption of glycerol as probably, some of the accumulated xylitol was contributing to cell growth after exhaustion of glycerol. More work is required to decode this, however, results are in agreement with those of Ledesma-Amaro et al. [19]. The idea of the resting cell experiment was to investigate the reusability of the cell biocatalysts repeatedly, which will improve the bioprocess economics. Somehow, the results were not very encouraging. We suspect that it could be due to disrupted supply of reduced pyridine nucleotides and in future studies the biotransformation medium could be supplemented with co-substrate at regular interval for uninterrupted supply of a redox cofactor to carry out the reduction reaction [14].

The final step of recovery and purification of a product in a bioprocess is challenging and determines the feasibility of the process, especially for products synthesized from crude renewable sources. Its complexity depends on the nature of product and composition of the fermentation broth. The information on xylitol recovery from fermentation broths is scarce in literature [13]. This study reaffirmed that a low cost easily available adsorbent like activated charcoal could efficiently remove the impurities and simultaneously decolorized the xylitol rich fermentation broth as reviewed previously [34]. Earlier, Gurgel et al. [47] recovered ~ 80% of xylitol when 25 g of activated charcoal was added to 100 mL fermentation broth with pH adjusted to 6.0 and incubated at 80 °C for an hour. This result is inferior to the present results where lesser quantity of activated charcoal (25% versus 5%) for xylitol purification was used. However, when different types of activated carbon were evaluated, Wei et al. [48] observed that 4% M1 type of activated carbon was able to recover 95% xylitol with 96% decolourization. Thus, there is a scope of screening different forms of activated carbon for further improving the xylitol recovery. The xylitol recovery further reduced in the final step of alcohol precipitation step and dropped to 35–40%. The same strategy used previously resulted in the recovery of 43.7% xylitol [49]. When molecular dynamic computer simulations were carried out for the binary mixture of polyols and water, Politi et al. [50] suggested that water forms an average number of 1.3 H-bonds with xylitol. However, when excess of ethanol was used for precipitation of xylitol, there is a likelihood that it not only reduced the affinity of water to form H-bond with xylitol leading to its partial precipitation but simultaneously also formed H-bond with xylitol resulting in its poor recoveries.

Biological production of xylitol has been studied for decades using a number of organisms including bacteria, yeasts and fungi. In general, the bioproduction of xylitol by bacteria and fungi have lower performance in comparison to yeasts. Among yeasts, *Candida* species are the most researched and best organisms for bioproduction of xylitol, yielding high conversion rates and productivities [51, 52]. Table 4 compares the results obtained in the current work with the literature reports and large variation exists in terms of xylitol titer, yield and productivity. Most of the organisms shown in Table 4 have active xylose assimilation pathway and demonstrated incredible xylitol producing ability using xylose as a sole carbon source. Some of them have been metabolically engineered to design hyper xylitol producing cell factories. Su et al. [53] designed recombinant *Escherichia coli* by xylose pathway engineering and eliminating carbon catabolite repression to allow simultaneous utilization of glucose and xylose. This recombinant strain yielded 172.4 g/L in 110 h. Majority of the reports including current study suffers from low volumetric productivities restricting their application in a broader perspective. The use of resting cells, membrane bioreactors and fed-batch fermentation has resulted in remarkable improvement in productivities in range of 3–12 g/L h [54–56].

**Table 4:** Bioproduction of xylitol by different microorganisms

Microorganism	Xylitol			Reference
	Titer (g/L)	Yield (g/g)	Productivity (g/L h)	
*Corynebacterium* sp. B-4247	48	0.48	2.0	[57]
*Corynebacterium glutamicum*	166	–	7.9	[38]
*Escherichia coli*	172.4	–	1.57	[53]
*Candida guilliermondii* FTI-20037	77.2	0.74	–	[58]
*Candida tropicalis*	131	0.87	2.91	[59]
*Candida boidinii* NRRL Y-17213	53.1	0.47	0.16	[60]
*Candida* sp.559-9	173	0.87	1.44	[40]
*Candida athensensis* SB18	256.5	0.87	0.97	[61]
*Candida tropicalis* CCTCC M2012462	38.8	0.70	0.46	[62]
*Candida tropicalis* SS2	220	0.93	3.3	[55]
*Candida tropicalis* KCTC 10457	182	0.85	12.0	[54]
*Pichia* sp. YS 54	25	0.76	0.50	[63]
*Debaryomyces hansenii* UFV-170	76.6	0.73	0.37	[64]
*Hansunela anomala* NCAIM Y.01499	21.7	0.47	0.23	[65]
*Kluyveromyces marxianus* CCA510	12.3	0.50	0.17	[66]
*Kluyveromyces marxianus* YZJ015	71.4	0.89	4.43	[56]
*Hansenula polymorpha*	58	0.62	0.60	[67]
*Saccharomyces cerevisae*	19	0.95	~0.20	[69]
*Aspergillus niger* PY11	1.14	0.101	–	[68]
*Y. lipolytica*^a^	53.2	0.97	0.32	This study
*Y. lipolytica*^b^	50.5	0.92	0.30	This study

^a^ Pure glycerol as co-substrate

^b^ Crude glycerol as co-substrate

It is important to discuss here the report by Hallborn et al. [69] where xylitol was produced from recombinant *Saccharomyces cerevisiae*. *S. cerevisiae* and *Y. lipolytica* are two very different organisms; former is a conventional yeast and later is non-conventional oleaginous yeast. *S. cerevisiae*, exhibits a clear preference for glucose and is well adapted to assimilate it. The ability of *S. cerevisiae* to utilize glycerol is limited and therefore, the yeast is not an attractive cell factory to use glycerol, a major industrial byproduct. Contrary to *S. cerevisiae*, *Y. lipolytica* prefer glycerol over glucose and exhibits higher growth rates on glycerol than glucose [16–18]. Hallborn et al. designed the recombinant strain of *S. cerevisiae* by overexpressing xylose reductase to enable xylitol production in the yeast whereas *Y. lipolytica* used in the current work cannot grow on xylose but has the natural ability to transform of xylose to xylitol [19]. The cell biomass of recombinant *S. cerevisiae* was grown on glucose for the biotransformation of xylose to xylitol. Though the xylitol yield (g/g) obtained in both the studies were over 90%, the titer achieved in current work (53.2 g/L) was much higher than Hallborn et al. (19.0 g/L).

The *Y. lipolytica* strain used in the current work lacks the effective pentose pathway and require addition of co-substrate for growth. The competitive titer and yield near to the theoretical demonstrates the remarkable potential of *Y. lipolytica* for xylitol production. The results obtained are better than many of existing reports. The volumetric productivity is not competitive with the best xylitol producers, but we are hopeful that it can be improved to industrial level using metabolic and process engineering approaches as mentioned above. The other advantageous feature of *Y. lipolytica* is the limited ability to cause only mild, self-limiting infections which confer the GRAS status to the yeast [70]. The main application of xylitol is in food and pharmaceutical industries, but pathogenic behaviour of most of the promising xylitol producers impedes their commercialisation. For example, *Candida* sp are prolific pathogens and cause globally almost 90% of fungal infections. As a result, the use of *Candida* sp. are prohibited in food industries [39, 40, 71]. The high yield of xylitol achieved with *Y. lipolytica* along with its safe behaviour keep it in superior position and make it a promising microbial cell factory for xylitol production.

In the current study, the bioproduction of xylitol was coupled with two waste streams, CG, a major industrial by-product and xylose, the second major sugar present in the hemicellulosic fraction of biomass. The impurities present in CG limits its application in chemical industries as the refining costs offset the profit and because it has limited applications. For example, the large amount of CG generated in UK from biodiesel industries (Greenergy, Croda, Oil Works) has no proper use and is exported to Germany, Netherland and East Europe and sold at very low price (£40–£150/ton). Some small manufacturers are even paying for collection of CG [72]. However, if the CG could be used locally, then environmental and economic benefits may be gained. To the best of our knowledge, this is the first study where CG was used for xylitol production. The results obtained with PG as well as CG were consistent indicating a high level of tolerance by the yeast to the impurities in CG as no inhibition was observed during the course of fermentation. These results are in agreement with many other studies where *Y. lipolytica* has been cultured on CG for the production of organic acids such citric acid, succinic acid etc. [15, 73, 74].

## Conclusion

Xylitol is a platform chemical with vast commercial potential. This is the first detailed report of bioproduction of xylitol by *Y. lipolytica*. The current work demonstrates enormous potential of *Y. lipolytica* to convert xylose to xylitol with a yield near to the theoretical (> 90%). It produces similar concentrations of xylitol to some of the best xylitol producing organisms such as *Candida* strains. Moreover, it is a safe organism to use with GRAS status and exhibited high tolerance to CG and xylose. Employment of unconventional feedstocks as carbon sources is highly desirable for the economic viability of biorefineries and becomes a good destination for renewable carbon-rich wastes. The study demonstrated the feasibility of simultaneous valorisation of two major wastes, CG and xylose, which can be utilized as cheaper feedstocks. The strategy can be conducive towards development of a bioprocess as an alternative to the commercial chemical route and could support the sustainability of biodiesel industries/lignocellulosic biorefineries. More work is required to optimise the metabolic engineering and process scale-up to improve the economics of the bioprocess.

## Materials and methods

### Materials

All chemicals used in this study were of analytical grade and purchased from Sigma-Aldrich and Fisher scientific, unless stated otherwise. CG used was kindly provided by Greenergy, UK. The CG contained glycerol (72.8%), non-glycerine material (soaps, fatty acids, esters, salts, other organic byproducts) (5.7%), methanol (2.0%), water (12.2%) and ash (9.6%). The xylose (26.4 g/L) rich SCB hydrolysate was obtained from Nova Pangea Technologies, UK.

### Microorganism, culture maintenance and inoculum preparation

The current study made use of *Y. lipolytica* Po1t (Ura^+^, Leu^+^) derived from wild-type strain W29 (ATCC20460). The *Y. lipolytica* strain was preserved in 20% glycerol (v/v) at − 80 °C and maintained on a petri dish containing YPD agar medium (1% yeast extract, 2% Peptone, 2% Dextrose and 2% Agar) at pH 7.0 and 30 °C. The seed culture was grown in a 250 mL Erlenmeyer flask containing 50 mL YPD broth. The final pH of the medium prior to sterilization was adjusted to 7.0. Cultivation was carried out for 24 h at 30 °C on a rotary shaker at an agitation speed of 250 RPM.

### Submerged cultivations in shake flask

The fermentation medium had the following composition: (g/L) PG/CG/glucose, 20; xylose, 20; yeast nitrogen base (YNB), 1.7; NH_4_Cl, 1.5. The medium was prepared in 50 mM phosphate buffer. The initial pH was adjusted to 6.8 before inoculation by using 5 N NaOH. The submerged cultivations were carried out in 500 mL shake flasks containing 100 mL working volume. The flasks were inoculated with fresh inoculum at OD_600_ (optical density) of 0.1 and kept at 30 °C under constant shaking at 250 RPM on a rotary shaker (Excella 24, New Brunswick).

### Central composite design (CCD) and artificial neural network linked genetic algorithm (ANN-GA) for media optimization

The CCD was carried out, with the view of optimizing the variables and to give insight over the combined effect of four variables (xylose, YNB, NH_4_Cl and phosphate buffer) at constant glycerol concentration on maximizing the production of xylitol concentration. Design-Expert software (version 7.0) was used to develop CCD for four independent variables and five levels (Table 5). The total number of experiments (*N*) was based on Eq. (1)

**Table 5:** Experimental codes, range and levels of the independent variables used for central composite design (CCD) experiments

Independent variables	Units	Symbol code	Coded value				
			+α	−1	0	1	−α
Xylose	g/L	X_1_	5	20	35	50	65
YNB	% (w/v)	X_2_	0.05	0.2	0.35	0.5	0.65
NH_4_Cl	% (w/v)	X_3_	0.05	0.2	0.35	0.5	0.65
Phosphate buffer	mM	X_4_	2.5	35	67.5	100	132.5

1 $$N = 2^{k} + 2k + 6$$

where *k* is the number of independent variables. The experiment comprised 2 axial points and 6 replicates for centre points for the evaluation of pure error. The second-order polynomial for predicting the optimal levels was expressed according to the Eq. (2).

2 $${\text{Y}}_{i} = \beta_{0} + \mathop \sum \limits_{i = 1}^{k} \beta_{i} X_{i} + \mathop \sum \limits_{i = 1}^{k} \beta_{ii} X_{i}^{2} + \mathop \sum \limits_{i < j} \sum \beta_{ij} X_{i} X_{j} + \varepsilon$$

where, Y_i_ is the Predicted response; β_0_ β_i_, β_ij_, β_ii_ are constant and regression coefficients of the model, X_i_, X_j_ represent the independent variables in coded values and ε represents the error.

To further optimise the media components, the artificial neural network (ANN) methodology was adapted. ANN is biological inspired model, which mimics neural system and tends to optimize non-linear systems. Multi-layer perceptron method was incorporated, and training of the network was based on feed-forward back propagation method. The network architecture consisted of four input layers (xylose, YNB, NH_4_Cl, phosphate buffer), eight hidden layers and one output layer representing xylitol concentration. In the feed-forward training system, the data was channelized from input to output via., hidden layer, which was connected by parameters such as weights (w) and biases (b). Transfer functions such as tan sigmoid (*f1*: tansig) and Pure linear (*f2*: purelin) were situated between hidden and output layer, respectively. Tansig sums up weighted input including the biases, and the purelin carried out the linearization function for the output. The predicted output function is represented by the Eq. (3)

3 $$Y_{p} = f2\left[ {w^{0} \times f1 \times \left( {w^{H} \times input vector + b^{H} } \right) + b^{0} } \right]$$

where Y_p_ is the predicted response, w^o^, b^o^ and w^H^, b^H^ are weights and biases of the output and hidden layer, respectively. The network training was carried out by adapting Levenberg–Marquardt (LM) backpropagation algorithm, which calculates error function based on the difference between actual output and predicted output. The algorithm was trained repeatedly until subsequent minimisation in the error between the input and output layer is met [75]. Mean squared error (MSE) was used to calculate error function using Eq. (4).

4 $$MSE = \frac{1}{N}\mathop \sum \limits_{i = 1}^{N} \left( {Y_{a} - Y_{p} } \right)^{2}$$

where, Y_a_ is the actual output, Y_p_ is the predicted output and N is the number of data points. The simulation of the network was carried out by in built neural network toolbox of MATLAB (version 2010a).

Genetic algorithm (GA) is a heuristic method used to determine the global optimal solution for a non-linear problem and are independent of initial values; GA is often coupled with ANN to achieve precise optimization values. GA follows four steps to find a global solution. In the first step, initialization of the solution for the population takes place followed by fitness computation. The selected individual based on the fitness computation then undergoes crossing over and mutation, creating a new set of individuals [76, 77]. This process is repeated until a global optimum value is achieved.

The trained neural network model was used as a fitness function to further optimise the input space. The schematic representation of ANN-GA algorithm for optimisation of medium components to maximize xylitol production was shown in Additional file 1: Fig S1. The objective function of GA is given by Eq. 5:

5 $${\text{Maximize Y}} = {\text{f}}\left( {{\text{x}}, {\text{w}}} \right),\quad {\text{x}}_{\text{i}}^{\text{L}} \le {\text{x}}_{\text{i}} \le {\text{x}}_{\text{i}}^{\text{u}} , \;{\text{i}} = 1,2,3 \ldots {\text{P}}$$

where f is the objective function (ANN model), x denotes input vector, w denotes corresponding weight vector, Y refers to the xylitol experimental yield, X denotes operating conditions, P denotes number of input variables, x_i_^L^ & x_i_^U^ are lower and upper bounds of x_i_ fitness of each candidate solution.

### Model validation under shake flask conditions

The integration of CCD and ANN-GA predicted some crucial parameters and their concentrations, which could give optimum xylitol yields. Therefore, it became essential to validate the predicted values at shake flask level based on the global optimum values obtained by ANN-GA training. Shake flask studies were conducted with PG as co-substrate. However, simultaneously the efficacy of *Y. lipolytica* Po1t (Ura^+^ Leu^+^) using co-substrate combinations namely PG + xylose rich SCB hydrolysate and CG + pure xylose was also evaluated to assess the tolerance, utilization and biovalorization ability of the said strain for carbon sources derived from renewable feedstock.

### Batch cultivation in bioreactor

The batch experiments were performed in a 2.5 L bioreactor (Electrolab Bioreactors, UK) with 1.0 L working volume. The inoculum was prepared using optimised media and the optimum values of media components were as follows (g/L): PG/CG, 20; xylose, 55; YNB, 5.0; NH_4_Cl, 3.94; phosphate buffer, 132.5 mM. The starting pH was 6.8 and not controlled during the fermentation. The temperature and agitation speed were controlled at 30 °C and 250 RPM, respectively, while the aeration rate was maintained at 2.0 L/min for initial 48 h and then changed to 1.0 L/min for the rest of fermentation period.

### Biotransformation by resting cells

For active cells, *Y. lipolytica* was grown on optimised medium with PG in 500 mL flasks containing with 20% working volume. The temperature, pH and agitation speed were maintained at 30 °C, 6.8 and 250 RPM, respectively. For the second stage (biotransformation), the cells were harvested in the late exponential period (after 48 h) when the OD_600_ was somewhere between 20 and 25. Immediately after, the culture was centrifuged at 2800×*g* for 10 min, and the resulting pellet was washed with ice-cold 100 mM phosphate buffer (pH 7.0). The cells were resuspended in a bioconversion medium containing xylose (30, 70 and 100 g/L) in phosphate buffer (100 mM). The bioconversion experiments were carried out at 30 °C with freshly prepared biomass.

### Downstream processing of xylitol

The purification protocol for xylitol was performed according to Rivas et al. [78]. The 100 mL of spent fermentation broth was subjected to centrifugation at 20,000×*g* to separate the cells and the clarified broth was treated with 5% activated charcoal. The charcoal treated broth was precipitated by adding four volume of absolute ethanol and incubated at 4 °C for 1 h. After 1 h, the precipitates were removed by centrifuging the mixture at 4000×*g* for 10 min. The supernatant was vacuum concentrated at 40 °C. The concentrated sample and ethanol were mixed at a ratio of 1:4 and incubated at − 20 °C with slight agitation (50 RPM) until crystals were observed. To improve the crystallization about 1 g/L of xylitol was mixed with the concentrated sample.

### Analytical methods

The samples were withdrawn periodically and analysed for OD, pH, residual glycerol/glucose, xylose and xylitol. Cell growth was quantified by measuring the optical density at 600 nm wavelength in a 1 mm-path-length cuvette using a double beam spectrophotometer (Jenway 6310, UK). One unit of absorbance at 600 nm corresponded to a cell dry weight (CDW) of 0.21 g/L. The concentrations of glycerol, glucose, xylose and xylitol were measured by high performance liquid chromatography (Agilent Technologies 1200 series, USA). The supernatants, obtained by centrifugation of the culture samples at 10,000×*g* for 10 min, were filtered through a 0.22 µm PVDF membrane (Sartorious, Germany) and eluted using Rezex ROA-Organic Acid H + (Phenomenex, USA) column at 60 °C attached with refractive index detector (RID). The mobile phase and flow rate were 0.5 mM H_2_SO_4_ and 0.4 mL/min, respectively. All measurements were conducted in triplicates and the values were averaged. The standard deviation was not more than 10%.

## Supplementary information

**Additional file 1: Figure S1.** Schematic representation of ANN-GA for achieving the global optimum value for the maximization of xylitol concentration from *Y. lipolytica.***Table S1.** Statistical measures and performance of the ANN model for training, testing, validation and all data.

## Data Availability

All data generated and analysed during this study are included in this published article and its additional files.
