# Supplementary material for: Biovalorisation of crude glycerol and xylose into xylitol by oleaginous yeast Yarrowia lipolytica
Source: Microb Cell Fact. 2020 Jun 3;19:121. doi: 10.1186/s12934-020-01378-1 (PMC7271524; doi:10.1186/s12934-020-01378-1)
Supplement: Supplementary file 1 — Additional file 1: Figure S1. Schematic representation of ANN-GA for achieving the global optimum value for the maximization of xylitol concentration from Y. lipolytica.Table S1. Statistical measures and performance of the ANN model for training, testing, validation and all data. [file 12934_2020_1378_MOESM1_ESM.docx]

**Figure S1:** Schematic representation of ANN-GA for achieving the global optimum value for the maximization of xylitol concentration from *Y. lipolytica*.





**Table S1**: Statistical measures and performance of the ANN model for training, testing, validation and all data.

|  | **Sets** | **MSE** | **R^2^** |
| --- | --- | --- | --- |
| Training | 70% | 1.309 | 0.9457 |
| Validation | 15% | 12.605 | 0.9842 |
| Test | 15% | 26.709 | 0.9768 |
| Overall |  | 7.425 | 0.93887 |
